# Shoulder pain after laparoscopic antireflux surgery: a single-center, randomized, open-label trial

**DOI:** 10.1007/s00464-025-11939-3

**Published:** 2025-07-02

**Authors:** Sumeet K. Mittal, Andrés R. Latorre-Rodríguez, Ross M. Bremner

**Affiliations:** 1 Norton Thoracic Institute, St. Joseph’s Hospital and Medical Center, 500 W Thomas Road, Phoenix, AZ 85013 USA; 2 School of Medicine, Creighton University, Phoenix Health Sciences Campus, Phoenix, AZ USA; 3 Grupo de Investigación Clínica, Escuela de Medicina y Ciencias de la Salud, Universidad del Rosario, Bogota D.C., Colombia

**Keywords:** Hiatal hernia, Minimally invasive surgery, Shoulder pain, Analgesia, Postoperative pain, Perioperative care, Local anesthesia

## Abstract

**Background:**

Post-laparoscopic shoulder pain (PLSP) is a significant source of postoperative morbidity after hiatal hernia repair and antireflux surgery. We evaluated whether adjunctive pain relief strategies—instillation of 0.25% bupivacaine over the left hemidiaphragm or postoperative back massager use—reduced opioid use or pain severity after laparoscopic antireflux surgery (LARS).

**Methods:**

This single-center, randomized, open-label trial included patients who underwent elective primary LARS between May 2021 and April 2024. Participants were randomly assigned (1:1:1) to one of three groups: A, standard medication regimen; B, standard + intraoperative instillation of 0.25% bupivacaine over the left hemidiaphragm; or C, standard + back massager use. Primary outcomes included overall pain and postoperative use of opioids. The secondary outcomes were pain frequency and severity by anatomical location. Assessments were performed 1, 3, 6, 24, 72, and 168 h after surgery. This trial was registered (ClinicalTrials.gov: NCT04936711) and is now complete.

**Results:**

Forty-three patients were randomized to groups A (n = 16), B (n = 10), and C (n = 17). One had a postoperative complication, and four were excluded (missing data: n = 2, incorrect massager use: n = 2), leaving 38 per-protocol patients (A: 16, B: 9, C: 13). The median age was 66 years (IQR 57–69), median BMI 28.4 kg/m^2^ (IQR 25.5–31.2), and 68.4% were female. Incidence of PLSP within 7 days was 97.6%. Overall, 84.2% required opioids with the lowest use in group B (A: 100%, B: 55.6%, C: 84.6%; p = 0.014). Group B also had lower rates of PLSP at 1 h (A: 75%, B: 44.4%, C: 88.2%; p = 0.025) as well as any pain at 3 h (A: 81.3%, B: 66.7%, C: 100%; p = 0.049).

**Conclusions:**

PLSP is very common after LARS. Intraoperative bupivacaine instillation at the diaphragm reduced opioid use and pain severity in the immediate postoperative period, while back massagers provided no additional benefit.

**Supplementary Information:**

The online version contains supplementary material available at 10.1007/s00464-025-11939-3.

Minimally invasive laparoscopic surgery is widely accepted as the preferred approach for managing various abdominal conditions, including appendicitis, cholecystitis, and abdominal and hiatal hernias [1]. Compared to open surgery, laparoscopy offers several advantages, such as reduced wound size, decreased analgesia requirements, shorter hospital stays, less postoperative pain, and faster recovery [1, 2]. However, it is frequently associated with postoperative cervical and shoulder pain—complications that are relatively uncommon in open procedures [1, 3–5].

Although the incidence of post-laparoscopic shoulder pain (PLSP) is up to 80% on the first postoperative day, its precise etiology remains unclear [1, 4]. It has been proposed that pneumoperitoneum, caused by carbon dioxide insufflation, induces peritoneal stretching and irritation of the diaphragm and phrenic nerves, leading to referred pain in the shoulders, proximal upper limb, and cervical region [1, 6]. Other plausible secondary mechanisms include regional musculoskeletal tension resulting from patient positioning, frequent head hyperextension maneuvers during the procedure, and exposure to thermal changes (e.g., cold temperatures) in the operating room [7, 8].

Notably, shoulder pain may be more pronounced after laparoscopic antireflux surgery (LARS) and other foregut procedures, where extensive manipulation of mediastinal and abdominal structures can increase the risk, severity, and frequency of PLSP [9]. Contributing factors likely include mechanical traction on blood vessels and nerves as well as surgical manipulation at the diaphragmatic crura (e.g., suturing, mesh reinforcement, relaxing incisions) [9]. These interventions may facilitate the release of local inflammatory mediators (i.e., chemokines), further exacerbating inflammation and phrenic nerve irritation.

Several multimodal pharmacological strategies (e.g., intraperitoneal anesthesia, intrathecal administration of anesthetics) [10–12] as well as non-pharmacological approaches (e.g., low-pressure carbon dioxide pneumoperitoneum, Hemovac drainage, shoulder massage, pulmonary recruitment maneuvers, intraperitoneal drainage, and intraperitoneal saline irrigation) [13–18] have been explored to mitigate PLSP and reduce opioid-derived medication use. However, most of these interventions have shown inconsistent efficacy or face barriers (e.g., scalability, costs, safety profile) to widespread implementation in surgical practice. Therefore, we aimed to evaluate the efficacy of two easily implementable and inexpensive adjunctive strategies for PLSP relief: (i) intraoperative local anesthetic instillation over the left hemidiaphragm (targeting phrenic nerve irritation mechanisms) and (ii) postoperative use of a commercially available back massager after primary LARS (targeting musculoskeletal tension mechanisms).

## Methods

### Ethics statement

The Institutional Review Board of St. Joseph’s Hospital and Medical Center, Phoenix, AZ, approved this study (PHX-21-500-157-50-18, approval date: 28-Apr-2021). All individuals provided written informed before enrollment in the study. Personal identifiers were removed, and the data were anonymously analyzed. Good practice guidelines, according to the 2013 Helsinki Declaration, were followed throughout the study. The Consolidated Standards of Reporting Trials (CONSORT) 2010 statement and checklist were followed to ensure transparency and overall quality of the manuscript (Supplementary Material S1).

### Study design, population, and recruitment

This single-center, randomized, open-label trial was registered at ClinicalTrials.gov (NCT04936711). Briefly, two adjunctive interventions (i.e., intraoperative instillation of 30 mL of 0.25% bupivacaine over the left hemidiaphragm or postoperative use of a commercially available back massager) to standard treatment for postoperative pain control were compared among patients who underwent elective LARS between May 2021 and April 2024 at a tertiary regional medical center in the southwestern United States of America (St. Joseph’s Hospital and Medical Center, Phoenix, AZ). Eligible patients were those (i) undergoing primary LARS, (ii) aged ≥ 18 years, (iii) with an American Society of Anesthesiologists (ASA) physical status classification score of 2. Main exclusion and discontinuation criteria were (i) age < 18 years, (ii) redo-procedures, (iii) use of open surgical approach or conversion, (iv) history of major psychiatric disorders (e.g., depression, anxiety, somatic symptom disorder), (v) history of chronic musculoskeletal pain, (vi) current or past chronic use of pain relief medications (e.g., opioids, antispasmodics, non-steroidal anti-inflammatory drugs) for any purpose, (vii) postoperative complications (Clavien-Dindo grade > I), and (viii) allergy to any component of the standard pain medication regimen.

### Surgical technique and perioperative care

All procedures were conducted by an experienced foregut surgeon. Briefly, the surgical technique for LARS was standardized and included: (i) hernia sac excision, (ii) esophageal and mediastinal mobilization, (iii) high mediastinal dissection, (iv) cruroplasty (using simple interrupted posterior, anterolateral, and anteromedial non-absorbable sutures), and (v) a partial (Toupet) fundoplication, with both limbs of the wrap anchored to the crura. Notably, no gastropexies or additional procedures (e.g., Collis gastroplasty) were performed. Throughout all procedures, the intraperitoneal pressure was monitored with a 12–15 mmHg target.

General anesthesia was provided throughout the surgery. Intravenous premedication included midazolam (0.3 mg/kg), fentanyl (1.5–2 ug/kg), and lidocaine (1 mg/kg), while induction was completed with propofol (1.5–2 mg/kg) and rocuronium (0.5–0.6 mg/kg). Maintenance was achieved with inhaled sevoflurane and oxygen (concentration of 0.5–2.5%, adjusted for age). Anesthesia adjustments were made at the discretion of the anesthesiologists. As nausea prophylaxis, a single dose of intravenous dexamethasone (8 mg) was provided at the beginning of the procedure, and ondansetron (4 mg) was provided at skin closure.

After surgery, all patients were transferred to the post-anesthesia care unit, where a nursing team monitored hemodynamic and oxygenation parameters. Once fully recovered from anesthesia, they were transferred to the surgical floor. If a nasogastric tube was placed intraoperatively, it was removed once the patient was free of nausea and a clear liquid diet was started. After surgery, nausea was routinely managed with oral (e.g., metoclopramide) and supplemental intravenous (e.g., ondansetron) antiemetics as needed. On postoperative day 1, if no complications were identified, patients were discharged after 24–36 h of in-hospital observation. All study-related data were recorded by a dedicated research assistant (see Time points and data collection section).

### Study groups and interventions

#### Group A (standard medication regimen)

This group serves as control. Patients received a standard postoperative pain management regimen, which included a local anesthetic at the port incision sites before skin closure (0.5% bupivacaine HCL with epinephrine [30 mL]) and oral medications (i.e., muscle relaxant [e.g., methocarbamol 1 g QID] and/or analgesics [i.e., acetaminophen 650 mg QID]). Opioid-derived pain treatment was available as required for breakthrough pain with oral oxycodone (5–10 mg elixir every 4 h PRN) or intravenous morphine (2 mg every 2 h or PRN if NPO).

#### Group B (standard medication regimen + local anesthetic instillation over the diaphragm)

In addition to the standard medication regimen, subjects within this group received a single 30 mL dose of intraperitoneal anesthetic (0.25% bupivacaine hydrochloride without epinephrine [i.e., 75 mg/30 mL]), which was directly instilled over the left hemidiaphragm, suprahepatic region, and falciform ligament using a disposable, plain, long blunt-tip applicator at the end of the procedure (i.e., before trocar removal).

#### Group C (standard medication regimen + commercial back massager use)

In addition to the standard medication regimen, subjects allocated to this group received a commercially available back massager device (RESTECK Shiatsu Kneading Shoulder & Neck Massager, Fujian, China) immediately after anesthesia recovery. They were instructed to use the massager in a semi-Fowler position at medium intensity with the heater function activated, covering both shoulders and neck. The device was to be used every 2 h for at least 15 min on the first postoperative day, then every 4 h until 72 h after surgery, and thereafter as needed. Upon discharge, patients were required to maintain a written diary documenting their device usage until postoperative day 7.

### Randomization and blinding

All patients were randomized (1:1:1) into one of three groups: group A (standard medication regimen), group B (standard medication regimen and anesthetic instillation over the diaphragm), or group C (standard medication regimen and back massager use), using a single computer-generated sequence of random assignments. Randomization was performed by an independent research assistant immediately before surgery using REDCap (Research Electronic Data Capture) and the operating surgeon was informed of the allocation group after anesthesia induction. Because of obvious differences across the procedures, the study was open-label.

### Time points and data collection

The study time points included a preoperative (baseline) assessment and postoperative follow-ups at 1, 3, and 6 h (± 30 min), 24 and 72 h (± 12 h), and 7 days (± 1 day) after surgical site closure. Data were collected through questionnaires documenting pain location and severity using a visual analog scale (VAS), where 0 represents no pain and 10 indicates the worst imaginable pain. An intervention control log was also used to track analgesic use.

During hospitalization (1, 3, 6, and 24 h follow-ups), medication and massager use were recorded in the patient’s medical records, and pain questionnaires were administered by a research assistant. Before discharge, patients received instructions on how to use and complete the intervention control log. To ensure data completeness and administer pain questionnaires at post-discharge follow-ups (72 h and 7 days after surgery), the research assistant contacted patients via phone.

### Study outcomes

The primary outcomes were postoperative opioid use for breakthrough pain (defined as any approved opioid taken for acute pain within the first 7 days after surgery) and overall pain severity at each specified time point. Secondary outcomes included pain frequency (i.e., the proportion of patients reporting pain) and pain severity at specific anatomical locations (abdominal, lower back, and shoulders) and the use of non-opioid adjuvant analgesics within 7 days after the surgery.

### Sample size and power analysis

Sampling was completed by convenience (i.e., consecutive individuals who met inclusion criteria were included). The original intended sample size was 90 subjects (i.e., 30 in each group) to achieve a reasonable power of ~ 0.90, presuming a reduction in the proportion of postoperative opioid requirements by at least 40% in groups B or C. The calculation was based on two-sided tests at a 0.05 level of significance and considering previous studies reporting that up to 90% of patients will require the administration of opioids after surgical procedures such as ventral hernia repair, cholecystectomy, or appendectomy [19]. However, due to low participant interest and delays caused by the COVID-19 pandemic, which halted elective procedures, enrollment was stopped at 43 participants, therefore, a post-hoc power analysis was conducted.

### Data analysis

Descriptive statistics were used to summarize the data. Continuous variables are reported as median with interquartile range (IQR), and categorical variables as count with proportion. Missing data were observed only in categorical variables and were considered missing completely at random; thus, a ‘missing’ category was added where appropriate. Primary outcomes were analyzed using both per-protocol (PP) and intention-to-treat (ITT) populations, while secondary outcomes were analyzed only in the PP population. The normality of the data distribution was assessed using the Shapiro–Wilk test. Comparisons between groups were made using the Mann–Whitney U test or Kruskal–Wallis test for quantitative outcomes, as appropriate, and Fisher’s exact test for categorical outcomes. The risk of requiring breakthrough opioids for pain was estimated for each intervention group and expressed as relative risk (RR) with 95% confidence intervals. An alpha (α) level was set at 0.05, and significance values were adjusted using the Bonferroni correction for multiple comparisons. All analyses were performed using SPSS Statistics v29.0 (IBM, SPSS Inc., Chicago, IL, USA).

## Results

### Study participants and baseline characteristics

**Fig. 1:**
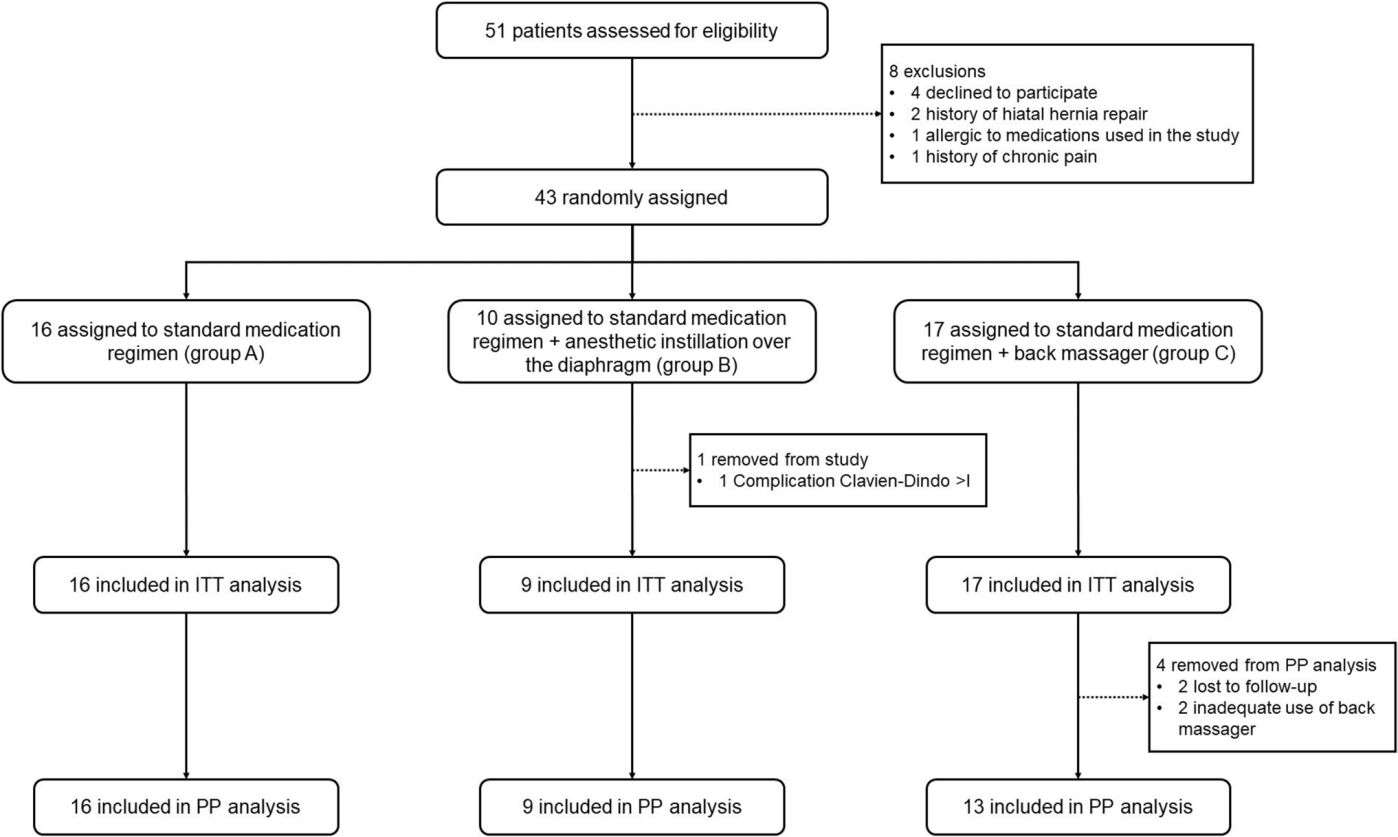
Study flow diagram. *ITT* intention to treat, *PP* per-protocol

A total of 51 patients were assessed for eligibility, with 8 excluded. The remaining 43 were randomly assigned to group A (n = 16), group B (n = 10), and group C (n = 17). One patient within group B was removed due to discontinuation criteria (i.e., postoperative complication), thus 42 patients were included in the ITT analysis. Four patients deviated from the protocol (lost to follow-up or incomplete treatment); thus, 38 patients (group A: 16, group B: 9, and group C: 13) were included in the PP analysis. The study flow diagram is presented in Fig. 1.

The baseline demographics and preoperative pain assessment for both ITT and PP populations are summarized in Table 1. Within the PP population, most patients were female (n = 26, 68.4%), with a median age of 66 years (IQR 57–69) and a median BMI of 28.4 kg/m^2^ (IQR 25.5–31.2). Age was significantly higher in group B than in group C (73 vs. 62 years, p < 0.05). Additionally, 7 patients (18.4%) reported experiencing some degree of non-chronic preoperative pain, with a median pain severity score of 2 (IQR 1–5). The pain locations included upper limb/shoulder (n = 2, 5.3%), head (n = 2, 5.3%), lower back (n = 2, 5.3%), and chest (n = 1, 2.6%).

**Table 1:** Baseline demographic characteristics and preoperative pain assessment for per-protocol and intention-to-treat populations

Per-protocol	PP group (n = 38)	Group A (n = 16)	Group B (n = 9)	Group C (n = 13)	p-Value
Demographic characteristics					
Sex, female	26 (68.4)	12 (75)	4 (44.4)	10 (76.9)	0.198
Age, years	66 [57–69]	67 [57.5–69]	73 [68–74]	62 [46–64]*	**0.039**
BMI, kg/m^2^	28.4 [25.5–31.2]	29.3 [28.2–32.3]	26.6 [25.4–28.5]	27.1 [23.6–30.5]	0.165
Preoperative pain assessment					
Any pain	7 (18.4)	3 (18.8)	0 (0)	4 (30.8)	0.188
Maximum pain severity^a^	2 [1–5]	1 [1–1]	–	4 [2.5–5.5]*	**0.028**
Pain location					
Abdomen	0 (0)	0 (0)	0 (0)	0 (0)	0.673
Chest	1 (2.6)	1 (6.3)	0 (0)	0 (0)	
Head	2 (5.3)	0 (0)	0 (0)	2 (15.4)	
Lower back	2 (5.3)	1 (6.3)	0 (0)	1 (7.7)	
Upper limb/shoulder	2 (5.3)	1 (6.3)	0 (0)	1 (7.7)	

Intention-to-treat	ITT group (n = 42)	Group A (n = 16)	Group B (n = 9)	Group C (n = 17)	p-Value
Demographic characteristics					
Sex, female	28 (66.7)	12 (75)	4 (44.4)	12 (70.6)	0.369
Age, years	64.5 [57–69]	67 [57.5–69]	73 [68–74]	62 [51–64]*	**0.028**
BMI, kg/m^2^	28.7 [26.5–32.4]	29.3 [28.2–32.3]	26.6 [25.4–28.5]	29.7 [25.5–32.4]	0.254
Preoperative pain assessment					
Any pain	8 (19)	3 (18.8)	0 (0)	5 (29.5)	0.257
Maximum pain severity^a^	1.5 [1–4]	1 [1–1]	–	3 [2–5]	0.057
Pain location					
Abdomen	0 (0)	0 (0)	0 (0)	0 (0)	0.795
Chest	1 (2.4)	1 (6.3)	0 (0)	0 (0)	
Head	2 (4.8)	0 (0)	0 (0)	2 (11.8)	
Lower back	2 (4.8)	1 (6.3)	0 (0)	1 (5.9)	
Upper limb/shoulder	3 (7.1)	1 (6.3)	0 (0)	2 (11.8)	

*ITT* intention-to-treat, *PP* per-protocol

All data presented as no. (%) or median [IQR]

Bold p-values represent statistical significance at < 0.05. Analyses were adjusted by the Bonferroni correction for multiple tests

*p-value < 0.05 compared to standard therapy (group A)

^a^Visual analog scale: 0 represents no pain and 10 indicates the worst imaginable pain

### Postoperative opioid use

**Fig. 2:**
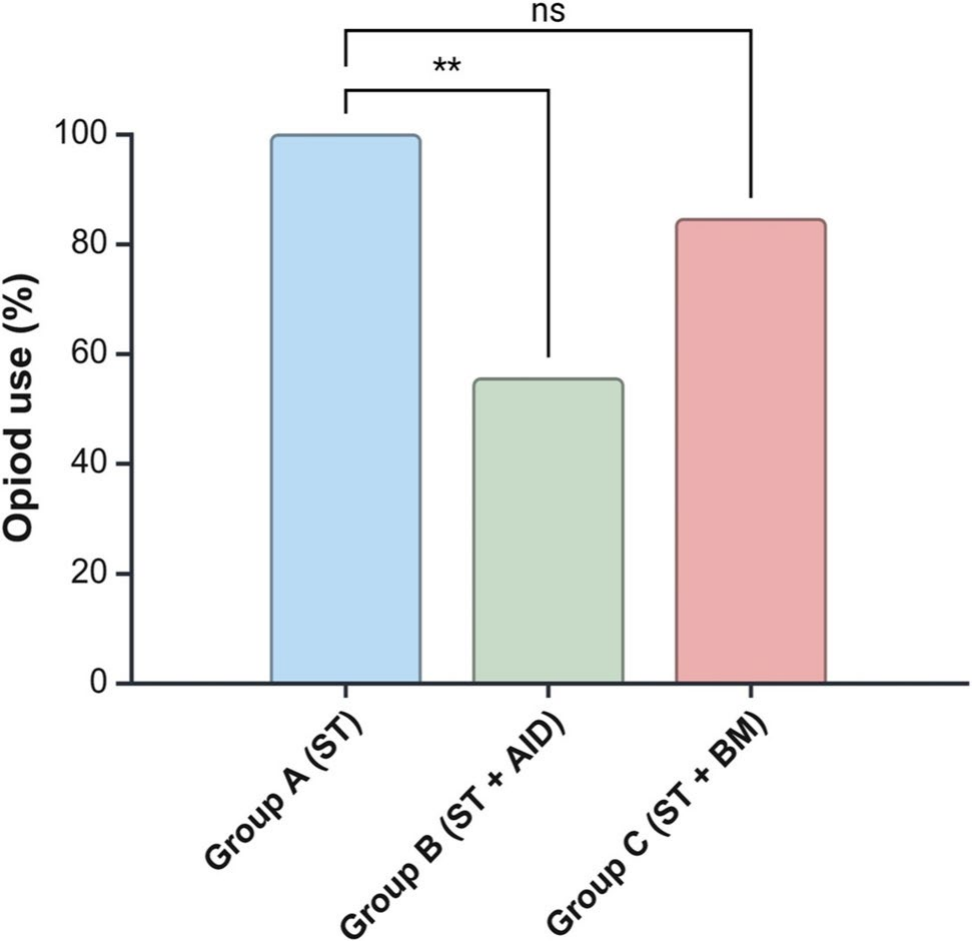
Opioid-derived medication use for populations according to allocation group (group A: 100%, group B: 55.6%, group C: 84.6%). *AID* anesthetic instillation over the diaphragm, *BM* back massager, *ST* standard therapy, *ns* no significant difference, **indicates a p-value < 0.01

Overall, 84.2% of patients among the PP population required treatment for breakthrough postoperative pain with opioid-derived medications within 7 days after surgery (group A: 16 [100%], group B: 5 [55.6%], and group C: 11 [84.6%]) (Fig. 2). Similarly, in the ITT population, the overall opioid usage was 81%. Of note, in both ITT and PP analyses, the use of intraperitoneal local anesthetic instilled over the diaphragm reduced the risk of needing opioids for breakthrough pain at any point (RR: 0.56 [95%CI 0.31–0.99], p = 0.049). Moreover, a slight decrease in opioid use (not statistically significant) was noted with the adjunctive use of the back massager (RR: 0.85 [95%CI 0.67–1.07], p = 0.157). Table 2 summarizes the frequency and type of opioid use across the groups.

**Table 2:** Opioid-derived medication use for per-protocol and intention-to-treat populations

Per-protocol	PP group (n = 38)	Group A (n = 16)	Group B (n = 9)	Group C (n = 13)	p-Value
Opioid use at any time	32 (84.2)	16 (100)	5 (55.6)*	11 (84.6)	**0.014**
Morphine use	21 (55.2)	10 (62.5)	4 (44.4)	7 (53.8)	0.679
Oxycodone use	29 (76.3)	15 (93.8)	5 (55.6)	9 (69.2)	0.074

Intention-to-treat	ITT group (n = 42)	Group A (n = 16)	Group B (n = 9)	Group C (n = 17)	p-Value
Opioid use at any time	34 (81)	16 (100)	5 (55.6)*	13 (76.5)	**0.009**
Morphine use	22 (52.4)	10 (62.5)	4 (44.4)	8 (47.1)	0.604
Oxycodone use	30 (71.4)	15 (93.8)	5 (55.6)*	10 (58.8)*	**0.047**

*ITT* intention-to-treat, *PP* per-protocol

All data presented as no. (%)

Bold p-values represent statistical significance at < 0.05. Analyses were adjusted by the Bonferroni correction for multiple tests

*p-value < 0.05 compared to standard therapy (group A)

### Non-opioid pain and adjuvant analgesics

In addition to the standard medication regimen, 10 patients (23.8%) reported the use of adjuvant non-opioid analgesics (group A: 2 [12.5%], group B: 3 [33.3%] and group C: 5 [29.4%]). However, there were no differences in the use of out-of-protocol adjuvant analgesics between the groups (p = 0.294).

### Overall postoperative pain severity

**Fig. 3:**
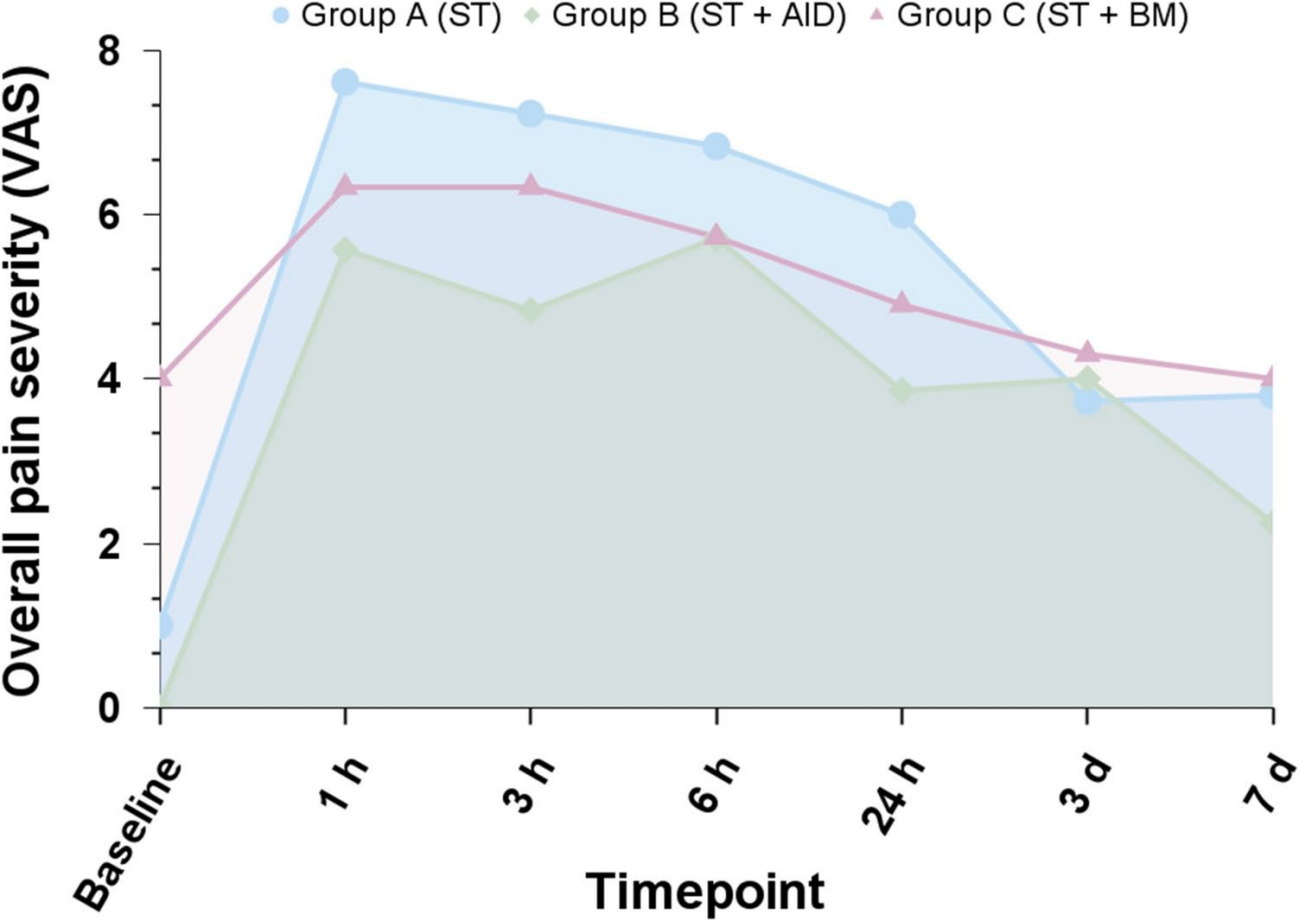
Mean overall pain severity over time by allocation group of the per-protocol population. *AID* anesthetic instillation over the diaphragm, *BM* back massager, *ST* standard therapy

Among the PP population, all but one patient (n = 37, 97.3%) reported any grade of postoperative pain during the study period. The maximum pain peak (i.e., severity) occurred between 1 and 3 h in all groups. Nevertheless, the proportion of patients reporting any pain 3 h after surgery was significantly lower in group B than in the other groups (Group A: 81.3%, Group B: 66.7%, and group C: 100%, p = 0.049). Moreover, a trend towards better pain control within the first 24 h was noted among group B. On postoperative days 3 and 7, the proportion of patients reporting pain and the severity of pain did not differ between groups. The patient-reported pain and pain severity trends among the PP population are presented in Fig. 3 and Table 3.

**Table 3:** Overall and specific anatomical location pain severity and frequency at each postoperative follow-up

Time point	1 h	3 h	6 h	24 h	3 days	7 days
Overall pain incidence and severity in the per-protocol population (n = 38)						
Group A, incidence:	13 (81.3)	13 (81.3)	12 (75)	13 (81.3)	11 (68.8)	5 (31.3)
Group A, severity:	8 [8–10]	8 [5–9]	7.5 [6–9]	5 [4–8)]	3 [2–5]	3 [2–4]
Group B, incidence:	7 (77.8)	6 (66.7)	7 (77.8)	7 (77.8)	7 (77.8)	5 (55.6)
Group B, severity:	5 [3–8]	4.5 [3–6]	5 [4–8]	3 [2–7]	4 [1–7]	2 [2–2.5]
Group C, incidence:	13 (100)	13 (100) δ	12 (92.3)	12 (92.3)	10 (76.9)	7 (53.8)
Group C, severity:	8 [3–8]	7 [5–8]	5.5 [4.5–8]	4 [3–6]	3.5 [3–5]	4 [2–5]
p-value, incidence:	0.446	**0.049**	0.756	0.821	0.901	0.243
p-value, severity:	0.124	0.182	0.340	0.177	0.663	0.192
Abdominal pain incidence and severity in the intention-to-treat population (n = 42)						
Group A, incidence:	3 (18.8)	2 (12.5)	2 (12.5)	4 (25)	3 (18.8)	5 (31.3)
Group A, severity:	7 [4–8]	5.5 [2–9]	4.5 [3–6]	4.5 [3.5–5]	1 [1–4]	3 [2–4]
Group B, incidence:	2 (22.2)	1 (11.1)	3 (33.3)	1 (11.1)	3 (33.3)	5 (55.6)
Group B, severity:	6.5 [3–10]	3	5 [1–5]	2	3 [1–5]	2 [2–2.5]
Group C, incidence:	7 (41.2)	9 (52.9)	10 (58.8)	8 (47.1)	4 (23.5)	8 (47.1)
Group C, severity:	2 [1–6]	2 [1–5]	4.5 [2–6]	4 [3–5]	2.5 [1.5–4.5]	4 [2–5]
p-value, incidence:	0.669	0.065	0.077	0.187	0.338	0.260
p-value, severity:	0.166	0.749	0.851	0.354	0.711	0.287
Lower back pain incidence and severity in the intention-to-treat population (n = 42)						
Group A, incidence:	7 (43.8)	4 (25)	5 (31.3)	7 (43.8)	3 (18.8)	2 (12.5)
Group A, severity:	8 [5–9]	9 [6–9.5]	7 [5–9]	3 [2–6]	2 [2–10]	2.5 [1–4]
Group B, incidence:	0 (0)	2 (22.2)	2 (22.2)	1 (11.1)	1 (11.1)	0 (0)
Group B, severity:	–	2.5 [2–3]	2.5 [1–4]	2	1	–
Group C, incidence:	11 (64.7)	11 (64.7)	9 (52.9)	4 (23.5)	2 (11.8)	0 (0)
Group C, severity:	7 [1–8]	2 [1–8]	3 [1–5]	4.5 [2.5–6]	3.5 [2–5]	–
p-value, incidence:	**0.011**	0.111	0.545	0.214	0.484	0.114
p-value, severity:	0.163	0.103	0.196	0.714	0.298	–
Shoulder pain incidence and severity* in the intention-to-treat population (n = 42)						
Group A, incidence:	12 (75)	13 (81.3)	11 (68.8)	10 (62.5)	10 (62.5)	2 (12.5)
Group A, severity:	8.5 [5.5–10]	8 [5–9]	6 [6–9]	6 [4–8]	2.5 [2–5]	5.5 [3–8]
Group B, incidence:	4 (44.4)	5 (55.6)	6 (66.7)	6 (66.7)	7 (77.8)	3 (33.3)
Group B, severity:	6 [4–7.5]	5 [4–6]	4.5 [3–5]	5 [2–7]	4 [1–7]	3 [1–3]
Group C, incidence:	15 (88.2)	15 (88.2)	15 (88.2)	13 (76.5)	8 (47.1)	7 (41.2)
Group C, severity:	8 [2–8]	7 [6–8]	6 [4–8]	5 [4–7]	5.5 [3-8]	5 [4–5]
p-value, incidence:	**0.025**	0.276	0.345	0.864	0.085	**0.015**
p-value, severity:	0.107	0.346	0.140	0.729	0.242	0.135

Group incidence data presented as no. (%). Group severity data presented as median [IQR] of patient-reported severity indicated on a visual analog scale from 0 (no pain) to 10 (worst imaginable pain)

Bold p-values represent statistical significance at < 0.05. Analyses were adjusted by the Bonferroni correction for multiple tests

**δ** indicates a p-value < 0.05 compared to group B

*Postlaparoscopic shoulder pain included patients reporting shoulder pain not only limited to the shoulder tip but also pain referred to the proximal upper limb and adjacent cervical region

### Localized postoperative pain

The proportion of patients in the ITT population reporting pain and pain severity over time according to the anatomical location are presented in Table 3.

**Fig. 4:**
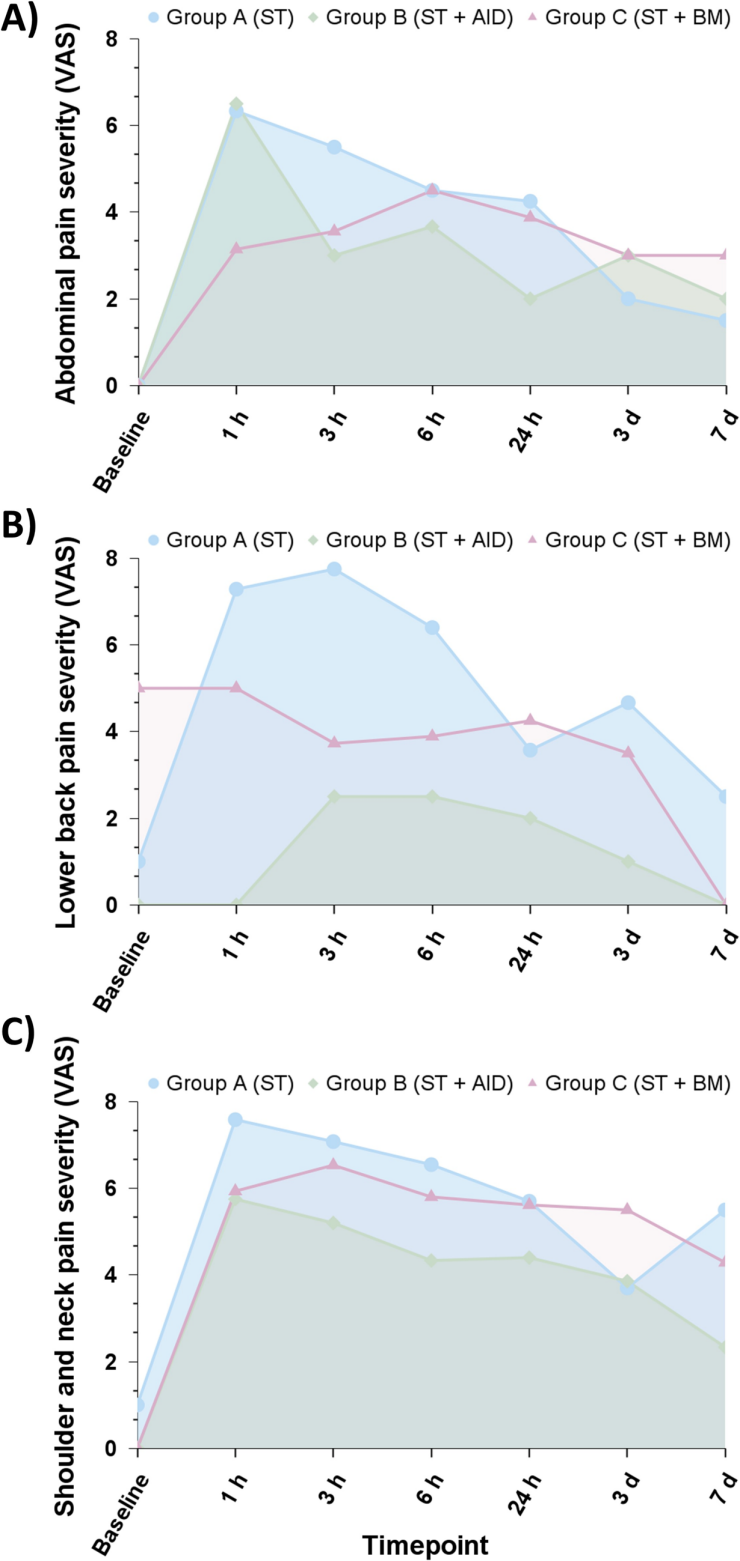
Mean pain severity over time among patients who reported specific anatomical location pain by allocation group. **A** Abdominal pain severity, **B** Lower back pain severity, and **C** Shoulder/neck pain severity. *AID* anesthetic instillation over the diaphragm; *BM* back massager; *ST* standard therapy

*Abdominal pain.* A total of 25 (59.5%) patients reported abdominal pain at any point within the first week after the procedure. The highest prevalence of abdominal pain (up to 58.8%) was noted between 3 and 6 h after surgery among group C; nevertheless, the highest severity was observed within 1 h after surgery in group A. Over time, the rates of abdominal pain and severity decreased similarly in all groups (Fig. 4A).

*Lower back pain.* Postoperative lower back pain was reported by approximately two-thirds of patients (n = 27, 64.3%). Interestingly, none of the patients within Group B reported lower back pain 1 h after surgery (Group A: 43.8%; Group B: 0% and Group C: 64.7%, p = 0.011), and the rates as well as the pain severity score remained lower in this group than the other groups (Fig. 4B). Further, although a higher proportion of patients reported lower back pain in group C compared to the control group, the median severity of pain tended to be lower.

*Postlaparoscopic shoulder pain.* A total of 41 patients (97.6%) reported postoperative shoulder pain and/or referred pain in adjacent areas (such as the neck or proximal upper limb), making this anatomical region the most commonly affected. Of these patients, 4 (9.8%) reported pain predominantly in the right shoulder area, whereas the rest reported similar pain severity in both shoulders (n = 22, 53.7%) or predominantly left shoulder pain (n = 15, 36.6%). Interestingly, one hour after surgery, a lower proportion of patients within Group B reported PLSP (Group A: 75%; Group B: 44.4% and Group C: 88.2%, p = 0.025), and PLSP severity trended lower in this group within the first 24 h after surgery. Further, there were no significant differences in pain rates or pain severity between groups on postoperative days 3 and 7 (Fig. 4C).

### Post-hoc power analysis

Considering the observed rates of opioid-derived medication consumption within 7 days after the procedure and a Type I error probability (α) of 0.05, an acceptable statistical power (~ 0.81) was achieved when comparing Group A (control) with Group B. However, for the other comparisons of the primary outcome (i.e., Group A vs. Group C or Group B vs. Group C), the analyses were somewhat underpowered (~ 0.37 and ~ 0.32, respectively).

## Discussion

Shoulder pain is a well-described source of postoperative morbidity after laparoscopic procedures, often necessitating additional analgesics (i.e., opioids) for breakthrough symptom relief [1, 19]. Previous studies have reported a PLSP incidence ranging from 35 to 80% after benign abdominal and gynecological procedures, which is primarily attributed to phrenic nerve irritation caused by carbon dioxide insufflation [4, 16, 17, 20]. However, foregut procedures, such as LARS, warrant special consideration, as they involve extensive manipulation of the crural diaphragm and both mediastinal and abdominal structures, potentially increasing the incidence and severity of shoulder pain. Although various pharmacological and non-pharmacological adjuvant strategies have been explored, findings have been inconsistent, and no universally accepted intervention is currently available for routine prevention of this complication [12, 17, 20].

We conducted a randomized, single-center, open-label trial with three arms to compare postoperative opioid consumption and the incidence and severity of localized pain by anatomical region. We found that: (i) the incidence of PLSP within 7 days after LARS was remarkably high, reaching up to 97.3%; (ii) instillation of bupivacaine hydrochloride over the left hemidiaphragm at the end of surgery significantly reduced the risk of requiring opioid-derived medications for pain control within the first 7 postoperative days compared to a control group (55.6% vs. 100%); and (iii) the use of a commercial back massager did not significantly decrease opioid use or reduce the frequency or severity of PLSP.

Compared to other laparoscopic abdominal or pelvic procedures, LARS appears to have a higher incidence of postoperative PLSP. Sarli et al. [16] and Gharaibeh et al. [20] reported a PLSP incidence of 21% and 60.5%, respectively, after laparoscopic cholecystectomy, and rates after gynecologic laparoscopic procedures range from 35 to 80% [21]. In our study almost all patients (i.e., 97.3%) reported PLSP in the early postoperative period. This finding supports the notion that, beyond diaphragmatic distension and subsequent phrenic nerve irritation caused by pneumoperitoneum, the type of surgery and the extent of mechanical manipulation of the diaphragm and mediastinum may play a significant role in increasing the frequency and severity of PLSP after foregut procedures.

This prospective study with frequent evaluation time points allowed us to characterize the presentation of PLSP specifically after LARS. In contrast to earlier reports on PLSP after cholecystectomy, where peak intensity was observed between 6 and 12 h after the procedure with a constant decrease until 48 h [16], our study found that after LARS, peak severity occurred between 1 and 3 h after the procedure, followed by a gradual decline up to day 7. This suggests that PLSP after LARS may present earlier and persist longer. This difference may be attributable not only to the nature of the procedure and the associated pathophysiological mechanisms discussed earlier but also to evolving anesthesia practices (i.e., more conservative, with reduced anesthesia facilitated by multimodal approaches and new monitoring tools) aiming for a faster patient recovery [22].

The intraperitoneal use of local anesthetics has been previously described. Nevertheless, their efficacy for PLSP after foregut procedures remains questionable. In 2015, McDermott et al. [11] reported results from a randomized controlled trial investigating the use of aerosolized intraperitoneal 1% ropivacaine (5 mL) in laparoscopic Nissen fundoplications or cholecystectomies. A total of 40 individuals were assigned to the intervention and 47 to placebo. Although no statistical significance was reached, the incidence of PLSP after 6 h at rest among patients treated with the intraperitoneal anesthetic was lower (52.5 vs. 71.7%, p = 0.066). The authors also noted relatively lower shoulder pain severity within the first 30 min after surgery (particularly for the subset of patients who underwent cholecystectomy); nevertheless, the effects beyond 24 h were not explored [11].

Further, a recent 2024 network meta-analysis by Dubey et al. [23] including 25 randomized controlled trials with 2401 participants, evaluated intraperitoneal amide local anesthetics (bupivacaine, lidocaine, levobupivacaine, and ropivacaine), their timing (pre- vs. post-pneumoperitoneum), and delivery method (aerosol vs. liquid) across various laparoscopic procedures. The authors reported that aerosolized bupivacaine administered before pneumoperitoneum deflation reduced postoperative opioid use compared to other methods, but did not impact pain scores or PLSP incidence within 24 h. Our findings align with those of Dubey et al. [23]. A plausible explanation for bupivacaine efficacy is its longer elimination half-life, allowing its effects to persist for several hours to days [24]. Although no adverse events were observed in our study, bupivacaine’s rapid vascular uptake and potential toxicity require careful consideration [24]. Therefore, it should be used with caution and at safe doses (≤ 2 mg/kg) [24].

On the other hand, the use of physical pain relief strategies, such as massage for PLSP, remains largely unexplored. To date, only a few randomized controlled trials including the present study, have evaluated this concept [25, 26]. In the randomized controlled trial by Mottahedi et al. [26], 138 laparoscopic cholecystectomy patients were randomized into three groups (n = 46 each). All received standard pharmacological treatment, with two groups additionally receiving either massage therapy or transcutaneous electrical nerve stimulation (i.e., TENS). Both interventions significantly reduced PLSP at 4, 8, and 12 h postoperatively, but no differences between the two were noted [26].

Similarly, Duran et al. [25] studied 60 laparoscopic cholecystectomy patients and found that those receiving a 10–15 min shoulder massage experienced reduced PLSP 30 min after the intervention compared to controls, but pain levels were similar between groups after 6 h. The differences between these findings and our results (which did not support physical intervention) may be due to (i) the tailored physical approach in other studies (i.e., hand massage by a professional therapist rather than using commercial massagers) and (ii) differences in outcome measurement timing, as prior studies assessed pain at time points after the intervention, whereas our design measured pain at pre-specified time points after surgery.

Although several non-pharmacological strategies have been shown to reduce PLSP (e.g., low-pressure carbon dioxide pneumoperitoneum, Hemovac drain placement, pulmonary recruitment maneuvers, opening and closing of intraperitoneal drainage, and intraperitoneal saline irrigation), we believe that standardizing and routinely implementing these procedures could increase costs and risks. Furthermore, these strategies may be technically challenging for healthcare providers (e.g., limited surgical working space and visibility with low-pressure carbon dioxide pneumoperitoneum) and somewhat cumbersome for patients (e.g., postoperative drains). Therefore, future studies evaluating large-scale implementation (i.e., generalizability) of alternative strategies, such as the use of intraperitoneal local anesthetics, are highly warranted.

Our study has multiple limitations. Despite several efforts and an extended recruitment period, we were unable to meet the enrollment goals established by the a priori sample size estimation, resulting in a relatively small sample size. Additionally, the use of simple randomization did not ensure a balanced distribution of participants and baseline characteristics between groups as initially expected. Due to the nature of the interventions, blinding was not feasible for either participants or investigators. Furthermore, a potential attrition bias arose, as all participants who did not complete follow-up assessments or received an “inadequate” intervention were allocated to the back massager group; however, for the primary outcome, both intention-to-treat and per-protocol analyses were conducted. Finally, although we evaluated multiple outcomes—opioid-derived medication use and the incidence and severity of postoperative pain—we acknowledge that only opioid consumption provides an objective measure of intervention efficacy. The assessment of pain using a visual analog scale is inherently subjective, potentially introducing an insensitivity bias due to individual variations in pain perception.

## Conclusion

Shoulder pain, not only of the shoulder tip but also referred to the proximal upper limb and adjacent cervical region (i.e., PLSP), is a common postoperative complication after LARS. Roughly 9 out of 10 patients in our study experienced some degree of PLSP within the first seven days after surgery, with the highest incidence occurring within the first 24 h. The intraperitoneal administration of local amide anesthetics (i.e., instilled over the left hemidiaphragm at the end of the procedure) reduced the need for opioid-derived medications in the immediate postoperative period. Therefore, future studies exploring the routine implementation of this strategy are highly warranted. Other physical strategies (i.e., use of commercial back massagers) targeting musculoskeletal tension mechanisms rather than phrenic nerve irritation may not provide additional benefits for PLSP management after LARS.

## Supplementary Information

Below is the link to the electronic supplementary material.

Supplementary file1 (DOCX 19 KB)

## Data Availability

The data analyzed in this study is allocated in a specific Research Electronic Data Capture (REDCap) database. It cannot be shared outside of those authorized as research staff per protocol. Access to this database requires IRB approval; if needed, direct to the corresponding author.
